# IOL use at Kilimanjaro Christian Medical Centre, Tanzania

**Published:** 2025-09-10

**Authors:** Wiliam Makupa

**Affiliations:** 1 Head of Department: Kilimanjaro Christian Medical Centre, Moshi, Tanzania.

**Internal audits of IOL consumption improved the availability of correct lens powers in a teaching hospital.**

**Figure:**
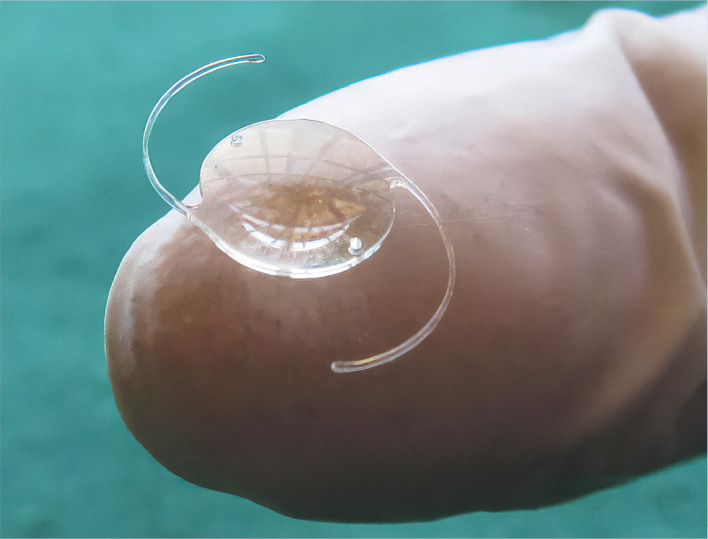
It is vital to have the correct IOL available.

© HEIKO PHILIPPIN. CC BY-NC-SA 4.0

The use of intraocular lenses (IOLs) at KCMC in Tanzania dates back to 1990, when we started using anterior chamber intraocular lenses. Posterior chamber intraocular lenses were introduced in 1992. By the year 2000, phacoemulsification services were available and foldable intraocular lenses were in common use.

Between 2011 and 2017, our department had been purchasing intraocular lenses based on overall numbers used and an estimate of the powers needed: a high volume of the most common powers (which we estimated to be 22D), and smaller quantities of higher and lower powers. We did not record or analyse the type or power of intraocular lenses used, which resulted in some higher-powered lenses expiring before they were used.

In 2017, we carried out our first internal audit of intraocular lens consumption based on lens type and dioptre. We found that the most common IOL used was 20D, not 22D as initially thought. We also found that the most common lens power we used in children was 25D.

This process continued to be improved annually until 2022, by which time a comprehensive picture had emerged of our average annual consumption of the three types of IOL we use most often – polymethylmethacrylate (PMMA), foldable hydrophilic, and hydrophobic acrylic – along their full dioptric ranges (from 1D to 30D).

“A comprehensive picture emerged of our average annual consumption of the three types of IOL we use most often.”

We found that the proportion of powers stayed the same, year on year, which makes it possible to estimate how many IOLs of each type and power we will need per year. We buy IOLs annually and – provided our finances allow it – we usually order 50% extra of each lens type and power, to ensure we don't run out.

When we do run out of a particular dioptric power, it is almost always as a result of neglecting to monitor the available stock. Stock monitoring is ideally done monthly, but it is challenging for us as our team has to manually tally the operating theatre stock with the main store stock; this makes it difficult to get real-time information of overall stock levels. However, as our store keeper and procurement officer gain more experience, this is less often a problem.

When a particular dioptric power intraocular lens is not available, and this is found out on short notice, the nearest dioptric power may be used, at the discretion of the surgeon; this is usually different by half a dioptre.

**The IOLs we use**

The IOLs we use most commonly in our setting are polymethylmethacrylate (PMMA) for small-incision cataract surgery (USD 10 per lens), followed by foldable hydrophilic acrylic IOLs (USD 25–35) and hydrophobic acrylic intraocular lenses (USD 40–90), for phacoemulsification. We also stock a few anterior chamber PMMA intraocular lenses, as well as scleral-fixated PMMA intraocular lenses and foldable hydrophilic three-piece intraocular lenses. Some of the foldable intraocular lenses come pre-loaded into a cartridge, making them easy to use, even for trainee surgeons. The foldable hydrophobic acrylic intraocular lenses are popular with our paediatric ophthalmology team and it's a great consolation that they give optimal results in children, considering the severe inflammation that can result from eye surgery in children.

We do not use toric or multifocal intraocular lenses. These decisions were made internally, by our ophthalmologists, after evaluating all aspects of using these products in our environment.

